# Genome‐Wide Analyses of Phosphate Transporters in Wild Rice (*Oryza brachyantha*) Revealed Their Evolution and Regulatory Roles in Phosphate Homeostasis

**DOI:** 10.1002/fsn3.70561

**Published:** 2025-07-02

**Authors:** Xusheng Zhao, Wai‐Shing Yung, Kejing Fan, Shengjie Chen, Hon‐Ming Lam

**Affiliations:** ^1^ School of Life Sciences and the State Key Laboratory of Agrobiotechnology The Chinese University of Hong Kong Hong Kong SAR P. R. China

**Keywords:** gene expression, *Oryza brachyantha*, phosphate starvation, phosphate transporter, phylogenetic analysis, wild rice

## Abstract

Recently, considerable progress has been made in understanding how cultivated rice adapts to phosphate (Pi) deficiency stress. However, little has been achieved in the genetic exploration of wild rice accessions, which are crucial for the development of new varieties adapted to Pi‐limited soils. In this study, we evaluated a collection of wild rice accessions for their phosphorus (P) absorption efficiencies using a hydroponic system, and identified *Oryza brachyantha*, a distant relative of the cultivated rice, as a promising candidate. Our investigation of phosphate transporters (PTs) in this wild species uncovered 31 *ObPT* genes across five families and analyzed their corresponding protein sequences. Phylogenetic and synteny analyses revealed that these ObPT proteins were highly conserved with their counterparts in cultivated rice ( *Oryza sativa* ), but six *OsPT* orthologs were lost from the *O. brachyantha* genome during its natural adaptation to the African savanna. Notably, only 24 out of 31 *ObPTs* were detectable by qRT‐PCR assays, and these genes showed different expression patterns in roots and leaves following prolonged Pi deprivation of up to 12 days. This dynamic expression pattern suggested an efficient reutilization of high P storage in roots and leaves for an extended period, potentially explaining the superior Pi utilization strategy by *O. brachyantha*. In conclusion, our study identified a wild rice species with high Pi uptake efficiency and provided novel insights into the genetic diversity and evolution of *PT* genes in a wild rice relative, illuminating their functions in Pi homeostasis.

## Graphical abstract

Oryza brachyantha, a wild rice species, was identified as highly efficient in phosphorus (P) uptake. Genome‐wide analysis revealed 31 phosphate transporter (ObPT) genes, with several ortholog losses and dynamic expression under Pi starvation. These findings highlight the evolutionary divergence and potential of wild rice in enhancing P use efficiency.

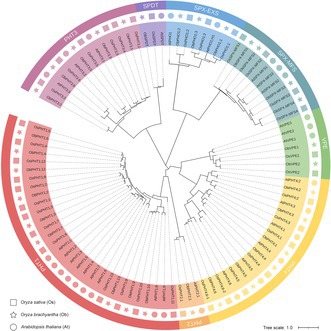

## 1 Introduction

Phosphorus (P) is an indispensable macronutrient for all living organisms, including plants, as it provides the integral backbone for the biosynthesis of nucleic acids, phospholipids, and the energy‐carrying molecule, ATP. Moreover, P is involved in important regulatory functions in metabolism and signal transduction through phosphorylation and protein activation (Raghothama 1999). Although P is abundant in the topsoil, it is commonly bound to soil particles or fixed in the form of P‐containing organic compounds, thereby limiting the bioavailability of P in the soil, especially for inorganic phosphate (Pi)—the major form of P assimilated by plant roots. Consequently, over half of the global arable land suffers from Pi deficiency, leading to compromised plant growth and reduced crop yields (Lynch 2011). This has prompted the extensive application of inefficient, non‐renewable, and polluting synthetic Pi fertilizers in agricultural practices to enhance crop productivity, especially in developing nations (Cordell et al. 2009). Therefore, we urgently need more sustainable alternative approaches to reducing the reliance of agriculture on Pi fertilizers while ensuring food security.

Recent advances have provided considerable insights into how both wild and cultivated plant species adapt to low‐Pi stress, which can be harnessed to breed or engineer crops with enhanced Pi acquisition or utilization efficiency (Dissanayaka et al. 2021). One strategy used by plants to liberate Pi from organic compounds in the soil is by secreting acid phosphatase and organic anions through the roots, as well as optimizing the root architecture to maximize the interactions with symbiotic microbes for the efficient Pi acquisition from the rhizosphere. Meanwhile, at the intercellular level, P is highly mobile within plants, and when a deficiency occurs, it may be translocated from old plant tissues to young and vigorously growing areas to enhance P utilization (Wang et al. 2010). These mechanisms are regulated by complex regulatory networks, involving Pi transporters (PTs) and other Pi starvation‐induced genes (PSIs).

Both low‐affinity and high‐affinity PTs in roots enable plants to absorb soluble Pi from the soil solution, where Pi is typically present at the micromolar level (Holford 1997; Lambers and Plaxton 2015; Lopez‐Arredondo et al. 2014). After entering the root symplast, Pi can be metabolized in the cytoplasm, or translocated by PTs into adjacent cells or subcellular compartments, where the vacuole acts as a temporary reservoir (Liu et al. 2014). These transporters are categorized into five families: H^+^‐coupled Phosphate Transporters (PHTs), SYG1/PHO81/XPR1‐ERD1/XPR1/SYG1 (SPX‐EXS/PHO), SPX‐Major Facility Superfamily (SPX‐MFS), Vacuolar Phosphate Efflux transporters (VPEs) and SULTR‐like Phosphorus Distribution Transporter (SPDT) (Wang, Chen, and Wu 2020; Xu et al. 2019). Based on the degrees of sequence identity and varied subcellular localizations, plant PHTs are further divided into the PHT1, PHT2, PHT3, and PHT4 subfamilies, whose members are localized to the plasma membrane, chloroplast, mitochondrion, and the Golgi membrane, respectively (Liu et al. 2011). As the largest and the most widely studied PT family, PHTs have been the primary focus of genome‐wide identification and characterization studies followed by detailed functional gene research (Ahmad et al. 2021; Lhamo et al. 2020; Liu et al. 2011; Wang, Xiao, et al. 2020; Yang et al. 2020). However, this approach has overlooked the relationships between PHTs and other PT families in phylogenetic and synteny analyses.

As one of the major staple foods worldwide, the production of rice ( *Oryza sativa* L.) needs to be further increased to ease the pressure from population growth and climate change. Like *Arabidopsis*, rice is a model plant that has been well studied in agricultural research. However, the genetic diversity of cultivated rice is limited compared to its wild relatives in the same genus. Over 20 wild species in the genus *Oryza* have been identified, exhibiting considerable genomic diversity, including varied genome sizes and the existence of AA, BB, CC, BBCC, CCDD, EE, FF, GG, KKLL, HHJJ, and HHKK genome types (*Sanchez* et al. 2013). In addition, these wild species are found in almost all tropical and subtropical regions, making them uniquely capable of surviving under a wide variety of environmental stresses and climate conditions. These widely distributed wild rice relatives constitute a huge genetic reservoir for crop improvement that can be exploited for breeding new rice varieties adapted to sub‐optimal Pi conditions. However, little effort has been made to conduct genetic research and germplasm exploitation in wild rice relatives, particularly in the context of P assimilation and re‐utilization.

In this study, we screened two cultivated rice subspecies and 12 wild rice species to assess their ability to accumulate P in root and shoot tissues. Among them, *Oryza brachyantha* displayed exceptional P accumulation under both normal and Pi‐depleted conditions, indicating a unique P use efficiency in this FF‐genome species. Unfortunately, there has been a lack of research on gene identification and functional validation since the reference genome of *O. brachyantha* was released a decade ago (Chen et al. 2013), so we conducted a genome‐wide identification and characterization of all putative PTs in *O. brachyantha*, to understand the molecular basis of its extraordinary P accumulation capacity and to elucidate the evolutionary history of *PT* genes in plants. Phylogenetic and synteny analyses revealed that all 31 putative *ObPTs* are highly conserved between cultivated rice and *O. brachyantha*, while 28.6% of the *OsPHT* orthologs were lost in the *O. brachyantha* genome during the adaptation by the latter to the African savanna. The temporal and spatial expression patterns of *ObPT* genes in roots and leaves were also determined under different Pi conditions. These findings offer insights into the Pi adaptation strategies employed by wild rice species, which will be useful for providing genetic resources for crop improvement.

## 2 Materials and Methods

### 2.1 Plant Materials and Growth Conditions

The Indica (Kasalath) and Japonica (Nipponbare) cultivars of rice ( *Oryza sativa* ) were used as controls in the screening experiment. All 12 accessions of wild rice species, including *O. brachyantha* (with one accession per species) were obtained from the National Institute of Genetics in Japan. Since wild rice seeds exhibit stronger dormancy than cultivated rice, the former were first scratched at the pericarp near the embryo to break the dormancy. Then the dehulled wild rice and intact cultivar seeds were germinated on PCR plates (with the bottom cut off and floating on MilliQ water) for 3 d in the dark at 30°C. Hydroponic experiments were performed using the Kimura B nutrient solution containing 540 μM MgSO_4_·7H_2_O, 360 μM (NH_4_)_2_SO_4_, 360 μM Ca(NO_3_)_2_·4H_2_O, 180 μM KNO_3_, 180 μM KH_2_PO_4_, 40 μM EDTA‐FeNa, 20 μM H_3_BO_3_, 12 μM MnCl_2_·4H_2_O, 0.8 μM ZnSO_4_·7H_2_O, 0.4 μM CuSO_4_·5H_2_O, and 0.04 μM (NH_4_)_6_Mo_7_O_24_·4H_2_O, with pH adjusted to 5.6 before use. For the Pi‐deficient treatment, 180 μM KH_2_PO_4_ was replaced by 90 μM K_2_SO_4_. Plant seedlings were grown in a growth chamber with a 14‐h light (600 μmol m^−2^ s^−1^, 30°C)/10‐h dark (25°C) photoperiod, and the relative humidity was maintained at approximately 70%. The nutrient solution was refreshed every 3 days.

### 2.2 Measurement of Total P and Soluble pi Concentrations

To evaluate the P accumulation ability and the P use efficiency of the cultivated and wild rice plants, the shoots and roots of five‐week‐old seedlings were sampled separately. After drying at 70°C in an oven for 72 h, plant samples were weighed and digested with concentrated nitric acid (60% [w/v]) at temperatures up to 180°C for 15 min in a microwave oven. The total P concentration was determined by an ICP‐OES (Perkin Elmer Optima 4300DV) following a previously described procedure (Khattak et al. 2015). The total P contents in roots and shoots were calculated by normalization to the corresponding dry weights.

To assess the P use status of *O. brachyantha* after Pi starvation, the roots and leaves of treated seedlings were collected separately to determine the soluble Pi concentration following a previously published procedure (Delhaize and Randall 1995; Deng et al. 2014; Gu et al. 2017), with modifications. Briefly, the weighed fresh tissues were homogenized with a mortar and pestle in liquid nitrogen. The inorganic phosphate (Pi) was extracted with 4 mL of 5% (v/v) sulfuric acid (5 M) solution. After centrifugation at 14,000× *g*, 200 μL of the supernatant was transferred and mixed with an 800‐μL aliquot of 1.5% (w/v) fresh ascorbic acid (pH 5.0) dissolved in the reaction buffer (2.8 M sulfuric acid, 10 g L^−1^ ammonium molybdate and 0.5 g L^−1^ antimony potassium tartrate). The mixture was incubated at 37°C for 30 min, and the absorbance at 880 nm was recorded by a spectrophotometer (SpectraMax Plus Absorbance Microplate Reader). The Pi concentration was calculated from a standard curve generated with varying concentrations of KH_2_PO_4_ and normalized by the fresh weight.

### 2.3 Identification and Phylogenetic Analysis of *ObPT* Genes

The full‐length genomic sequences, coding sequences (CDSs) and protein sequences of all PTs in *Arabidopsis thaliana* , *Oryza sativa* spp. *japonica*, and *Oryza brachyantha* were retrieved from the Ensembl Plants database (http://plants.ensembl.org) after BLASTP searches using 28 known AtPT and 37 OsPT proteins against the *O. brachyantha* genome (v1.4b) (*Chen* et al. 2013). Using the MEGA 11 software, 96 amino acid sequences were aligned in total, and phylogenetic analyses were conducted using the maximum‐likelihood method with 1000 bootstrap replicates (*Tamura* et al. 2021). The rough phylogenetic tree was annotated and polished using the online tool iTOL v5 (https://itol.embl.de/) (*Letunic and Bork* 2021). The putative ObPTs were named based on the corresponding OsPTs with the closest phylogenetic relationships.

### 2.4 Characterization of *ObPT* Genes and Associated Proteins

The exon‐intron structures of the putative *ObPT* genes were obtained using the GSDS online software (http://gsds.cbi.pku.edu.cn) (Hu et al. 2015). The physicochemical properties of the corresponding PT proteins, such as the theoretical isoelectric point (pI) and the molecular weight (MW), were calculated using the ProtParam tool on the ExPASy server (https://web.expasy.org/protparam/) (Gasteiger et al. 2005). The transmembrane segments (TMS) and subcellular localization of the ObPTs were predicted by the TMHMM Server (https://services.healthtech.dtu.dk/service.php?TMHMM‐2.0) (Krogh et al. 2001) and the Plant‐mPLoc server (http://www.csbio.sjtu.edu.cn/bioinf/plant‐multi/) (Chou and Shen 2010), respectively. The conserved motifs of ObPTs were analyzed by MEME (https://meme‐suite.org/meme /tools/meme) (Bailey et al. 2015) with default parameters and graphed by TBtools (Chen et al. 2020).

### 2.5 Chromosome Mapping and Syntenic Analysis

The length and gene density of each chromosome and the physical location of *ObPT* genes were retrieved from the genome annotation file of *O. brachyantha* (Chen et al. 2013). Subsequently, gene duplication events and collinearity relationships of *ObPT* genes within *O. brachyantha* and between *O. brachyantha* and *O. sativa* were analyzed using the One Step MCScanX tool in TBtools with E‐value < 1 × 10^−10^ (Chen et al. 2020). Then the information was visualized using an integrated diagram, which was drawn using the Advanced Circos tool in TBtools.

### 2.6 Quantitative Real‐Time PCR Analysis

Total RNA was isolated using the RNeasy Plant Mini Kit (Qiagen) according to the manufacturer's instructions. DNA removal and reverse transcription reactions are performed using the OneTaq RT‐PCR Kit (BioLabs). Real‐time PCR assays were performed using the QuantiNova SYBR Green RT‐PCR Kit (Qiagen) on a StepOnePlus Real‐Time PCR system according to the manufacturer's instructions (Applied Biosystem). Three biological replicates and four technical replicates were performed for each gene. The housekeeping gene *EF1⍺* (OB03G15790) from *O. brachyantha* was used as the internal control for all analyses. The primers for *ObEF1⍺* and *ObPT* genes are listed in Table S1.

### 2.7 Statistical Analysis

Statistics were performed using Student's *t*‐test. Differences were defined as statistically significant if *p* < 0.05.

## 3 Results

### 3.1 Performance of Wild Rice on P Uptake and Utilization

**FIGURE 1:**
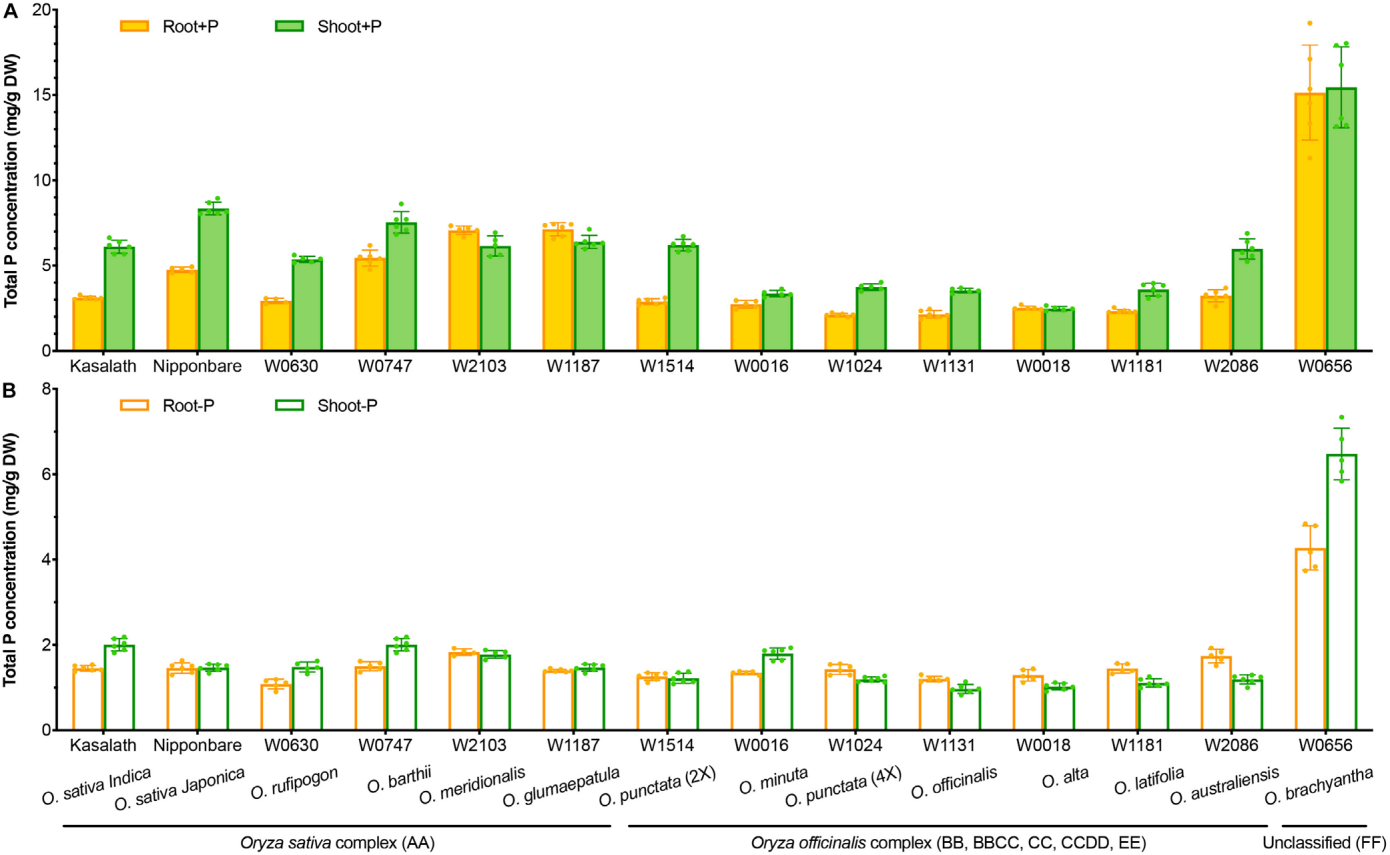
Total phosphorus (P) content of rice ( *Oryza sativa* ) and its wild relatives under inorganic phosphate (Pi)‐replete and Pi‐deficient conditions. Four‐week‐old seedlings of two cultivated subspecies of *O. sativa* (Kasalath and Nipponbare) and 12 wild species of rice (with one accession per species) were grown in a Pi‐replete (+P, 180 μM Pi) solution and then exposed to (A) +P (B) Pi‐deplete (−P, 0 μM Pi) environments for a week. Values represent means ± SD of six biological replicates, with each replicate being represented by a dot in the plot. DW, dry weight; Wxxxx, wild rice accessions.

Efficient P accumulation and utilization in crop plants are crucial parameters for agricultural production, especially under Pi‐deficient conditions (Cong et al. 2020). To assess P uptake and use efficiency among cultivated rice and its wild relatives, we conducted a phenotypic screening of 13 species spanning the AA to FF genome types within the genus *Oryza*, on their P accumulation in root and shoot tissues under Pi‐replete (+P) and Pi‐depleted (−P) treatments. Among the species screened, *O. brachyantha* (accession W0656) exhibited the highest total P concentration in roots (15.15 mg g^−1^ DW) and shoots (15.46 mg g^−1^ DW) under the +*P* condition, while the total P in other species ranged from 2.13 to 8.35 mg g^−1^ DW (Figure 1A). After Pi‐starvation for 1 week, the total P concentration of each species decreased sharply, by 32.7%–80.4% in roots and 52.8%–84.6% in shoots, while *O. brachyantha* still maintained the highest P content in all tissues despite a decrease of 74.7% and 58.1% in roots and shoots, respectively (Figure 1B). These results suggest that this wild rice species may possess a unique P usage strategy, enabling it to accumulate and store higher levels of P in cells to sustain growth during a prolonged fluctuation in Pi levels in the environment.

### 3.2 Identification and Characterization of Phosphate Transporters in *Oryza brachyantha*

In plants, Pi absorption and homeostasis highly depend on transport processes directly regulated by various PTs localized in cellular and subcellular membranes (Wang et al. 2017). To understand the molecular basis for the high P accumulation by *O. brachyantha*, we conducted a genome‐wide identification and characterization of all putative PTs in this wild rice species.

As shown in Table S2, a total of 31 *ObPT* genes were identified by blasting the protein sequences of known *Arabidopsis* AtPTs and *O. sativa* OsPTs against the *O. brachyantha* genome (Chen et al. 2013). These genes are distributed among the five *PT* families, including 21 *ObPHTs* (encompassing nine *ObPHT1s*, one *ObPHT2*, five *ObPHT3s* and six *ObPHT4s*), three *ObSPX‐EXSs*, four *ObSPX‐MFSs*, two *ObVPEs*, and one *ObSPDT*. The corresponding proteins contain from 351 (ObPHT3;5) to 897 (ObPHO1;3) amino acids (aa), with molecular weights (MW) between 37 and 101.6 kDa (Table 1 and Table S3). Their predicted isoelectric point (pI) values range from 4.86 to 10.42, with ObPHT1s typically having a pI around 8.75. According to the prediction of transmembrane helices using the HMMTOP tool in the Transport Classification Database (https://tcdb.org/progs/TMS.php) (Tusnady and Simon 2001), the number of transmembrane segments (TMS) in ObPT proteins varies from 3 to 18, suggesting all ObPTs are membrane‐bound, a necessary feature for facilitating Pi ion transport (Table 1).

**TABLE 1:** Properties of phosphate transporters identified from the *Oryza brachyantha* genome.

Clade	Protein name	Transcript ID	Gene position	Protein length (aa)	Isoelectric point	MW (kDa)	No. of TMS
PHT1	ObPHT1;2	Ob03G000312.1	Chr03:2362675‐2375057	769	8.93	84.7	18
	ObPHT1;3	Ob10G000666.1	Chr10:10611962‐10613608	510	8.94	55.4	12
	ObPHT1;4	Ob04G000209.1	Chr04:2644524‐2646131	535	8.48	58.7	12
	ObPHT1;5	Ob04G000208.1	Chr04:2626382‐2628010	542	8.48	59.3	12
	ObPHT1;7	Ob03G000231.1	Chr03:1689833‐1691401	522	8.63	56.4	12
	ObPHT1;9	Ob06G000923.1	Chr06:8123817‐8126181	571	8.77	70	12
	ObPHT1;10	Ob06G000924.1	Chr06:8128902‐8131501	574	8.97	61.5	12
	ObPHT1;12	Ob03G000311.1	Chr03:2357331‐2358944	537	8.8	58.4	12
	ObPHT1;13	Ob04G000211.1	Chr04:2669743‐2671966	556	8.75	61.1	13
PHT2	ObPHT2;1	Ob02G001466.1	Chr02:17078510‐17082085	569	9.57	59	13
PHT3	ObPHT3;1	Ob02G002229.1	Chr02:24589392‐24593491	365	9.3	38.6	8
	ObPHT3;2	Ob03G000931.1	Chr03:7359777‐7363015	373	8.77	39.9	6
	ObPHT3;3	Ob04G000851.1	Chr04:11588533‐11592698	361	9.18	37.9	5
	ObPHT3;5	Ob09G000695.1	Chr09:10745841‐10749027	351	8.87	37	3
	ObPHT3;6	Ob09G001116.1	Chr09:14675729‐14681709	392	9.47	42.6	5
PHT4	ObPHT4;1	Ob01G000885.1	Chr01:7711502‐7716397	544	8.85	58	11
	ObPHT4;2	Ob05G001213.2	Chr05:14519729‐14524477	532	9.49	55.9	12
	ObPHT4;3	Ob01G002592.1	Chr01:28115344‐28119073	520	10.42	55.9	11
	ObPHT4;4	Ob09G001198.4	Chr09:15346697‐15360363	656	9.26	72.3	11
	ObPHT4;5	Ob09G001126.2	Chr09:14799973‐14806698	506	5.96	54.2	10
	ObPHT4;6	Ob11G000407.1	Chr11:3146809‐3148053	414	9.71	44.9	11
SPX‐EXS	ObPHO1;1	Ob01G000059.1	Chr01:493343‐499546	793	9.24	91.1	5
	ObPHO1;2	Ob02G002414.1	Chr02:26404452‐26414085	806	9.52	90.6	7
	ObPHO1;3	Ob06G000976.1	Chr06:8970708‐8976056	897	9.11	101.6	7
SPX‐MFS	ObSPX‐MFS1	Ob04G001397.1	Chr04:16692010‐16699734	838	6.18	93	11
	ObSPX‐MFS2	Ob02G001835.1	Chr02:20821203‐20827334	707	5.71	79	10
	ObSPX‐MFS3	Ob06G000145.2	Chr06:950172‐957223	760	8.78	84.5	12
	ObSPX‐MFS4	Ob09G000977.1	Chr09:13403693‐13406382	638	4.86	68.7	10
VPE	ObVPE1	Ob04G001317.1	Chr04:15768175‐15772954	522	8.7	56.2	12
	ObVPE2	Ob08G000289.1	Chr08:2243395‐2246789	499	7.69	53.5	12
SPDT	ObSPDT	Ob06G000218.1	Chr06:1488456‐1493724	690	9.35	74.3	12

Abbreviation: TMS, transmembrane segment.

**FIGURE 2:**
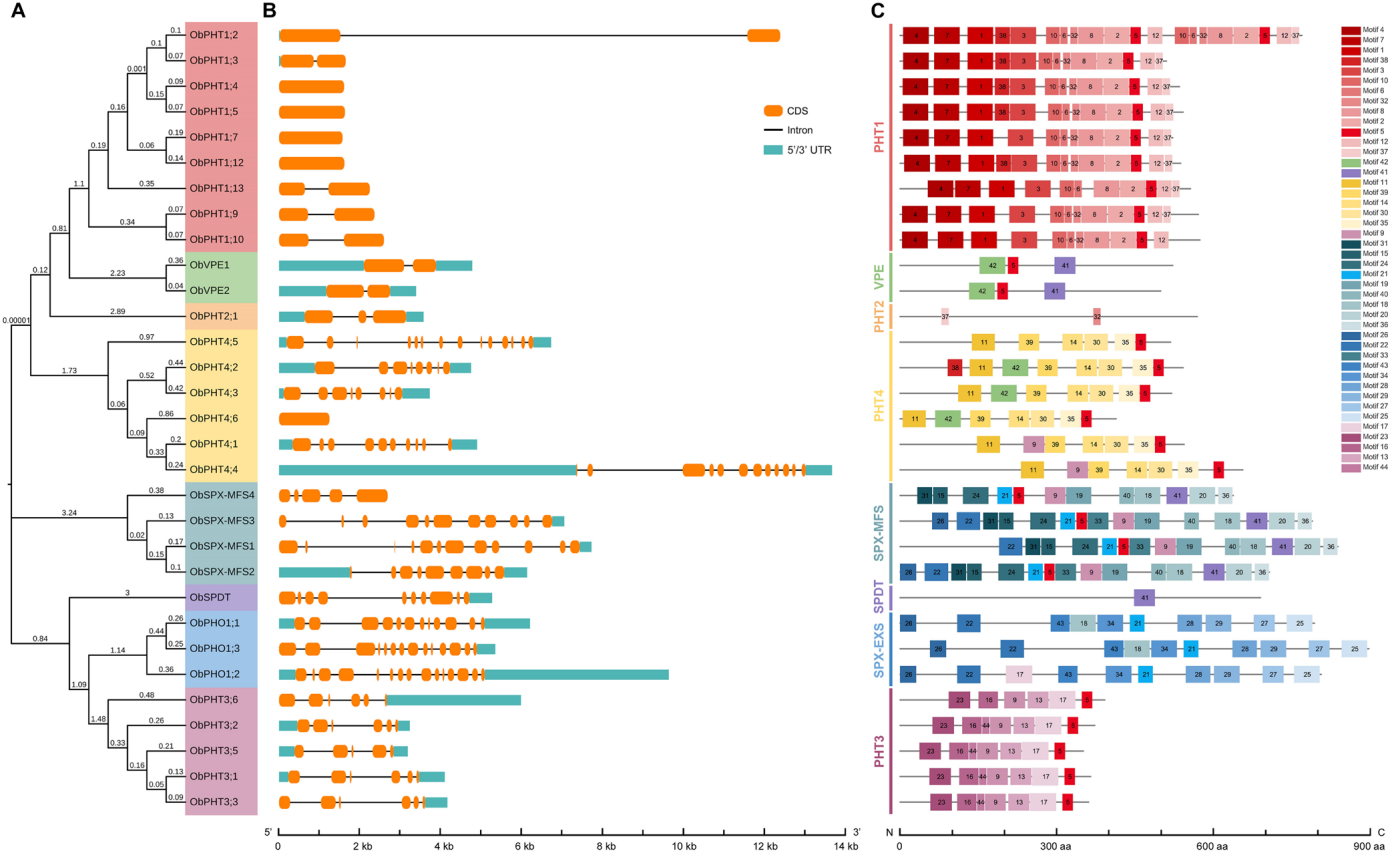
Phylogenetic and gene structure analyses of the phosphate transporter (PT) proteins in *Oryza brachyantha*. (A) An unrooted phylogenetic tree of ObPT proteins. The amino acid sequences of ObPTs were aligned and used for the construction of the phylogenetic tree. The proteins were sorted into eight clades, each highlighted in a distinct color. (B) The coding sequence (CDS)‐intron structures of the *ObPT* genes were determined by the alignments of CDSs with the corresponding genomic sequences. Orange bars indicate CDS fractions, while black lines represent introns, and turquoise bars indicate 5′ or 3′ untranslated regions (UTRs). (C) Forty‐four conserved motifs were identified using the MEME tool and identified by different colors. The *x*‐axes below indicate the lengths of gene fractions and protein motifs, respectively.

The phylogenetic tree of ObPT proteins illustrates the evolutionary relationships of these transporters in *O. brachyantha*, where ObPHT3s are closest to the ObPHO1 family but not to the other ObPHT subfamilies (Figure 2A). Gene structure analyses indicated that four *ObPHT1s* and *ObPHT4;6* lacked introns, while the coding sequences of other *ObPT* genes contained anywhere between one and 15 introns (Figure 2B). In addition to the similar exon‐intron structures of the encoding genes, *ObPT*s also share conserved motifs within the same clade (Figure 2C). For instance, *PHT1*s only contain one or two exons and share a similar amino acid sequence containing motifs 1–8, 10, and 12. These findings provided insights into the structural and functional characteristics of PTs *in O. brachyantha*.

### 3.3 Phylogenetic and Synteny Analyses of *ObPT* Genes

**FIGURE 3:**
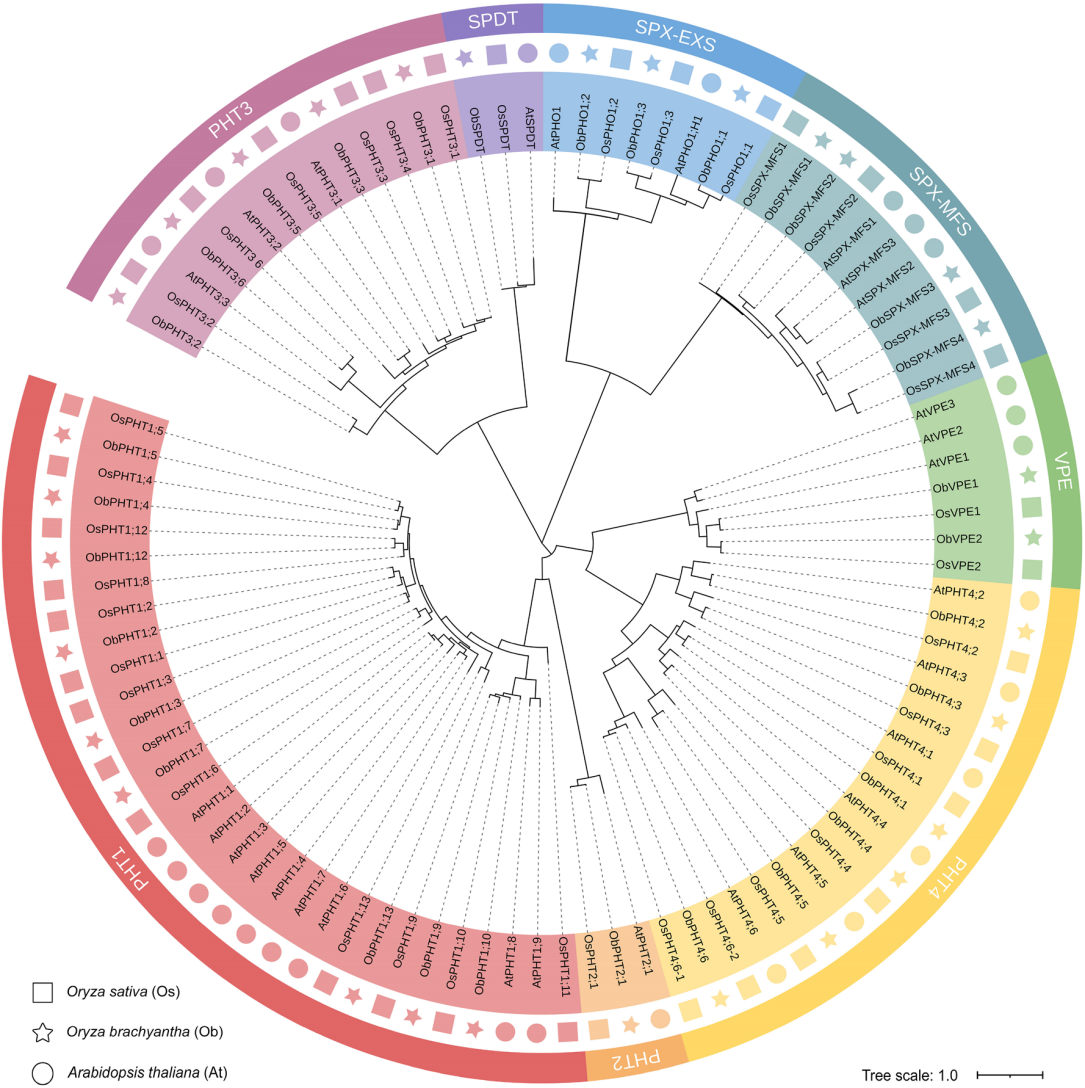
Phylogenetic analyses of phosphate transporters (PTs) in *Arabidopsis thaliana* (At), *Oryza sativa* (Os; cultivated rice) and *Oryza brachyantha* (Ob; a wild rice species). The phylogenetic tree was constructed via maximum‐likelihood method with 1000 bootstraps in the MEGA 11 program (*Tamura* et al. 2021) and annotated using iTOL v5 (*Letunic and Bork* 2021). Circles, squares, and stars next to the gene names represent the *PT* genes of *A. thaliana* , *O. sativa* , and *O. brachyantha*, respectively. The eight clades of *PT* genes are represented by the same eight colors as in Figure 2. All the gene names and locus IDs are provided in Table S2.

To investigate any possible evolutionary relationship among the 28 Arabidopsis AtPTs, 37 cultivated rice OsPTs, and 31 wild rice ObPTs, we constructed a phylogenetic tree based on their protein sequences using the maximum‐likelihood method in MEGA 11 (Tamura et al. 2021). All ObPTs could be paired with corresponding PT members from *O. sativa* (Figure 3), indicating a close phylogenetic relationship between the PTs in cultivated rice and those in its wild relative in general. Therefore, the ObPTs were named according to their OsPT orthologs. However, four OsPHT1s (including OsPHT1;1, OsPHT1;6, OsPHT1;8 and OsPHT1;11), OsPHT3;4, and OsPHT4;6–2 have no homolog in *O. brachyantha*, suggesting that these PTs (16.2% of all OsPTs) were lost from the *O. brachyantha* genome during natural adaptations to African habitats. In other words, about 19.4% of OsPTs were duplicated in cultivated rice during artificial selection and breeding. When clustering these five PT families together, PHT3s were observed to be more closely related to SPDTs than to other PHT subfamilies, and PHT4s were the closest to VPEs (Figure 3).

**FIGURE 4:**
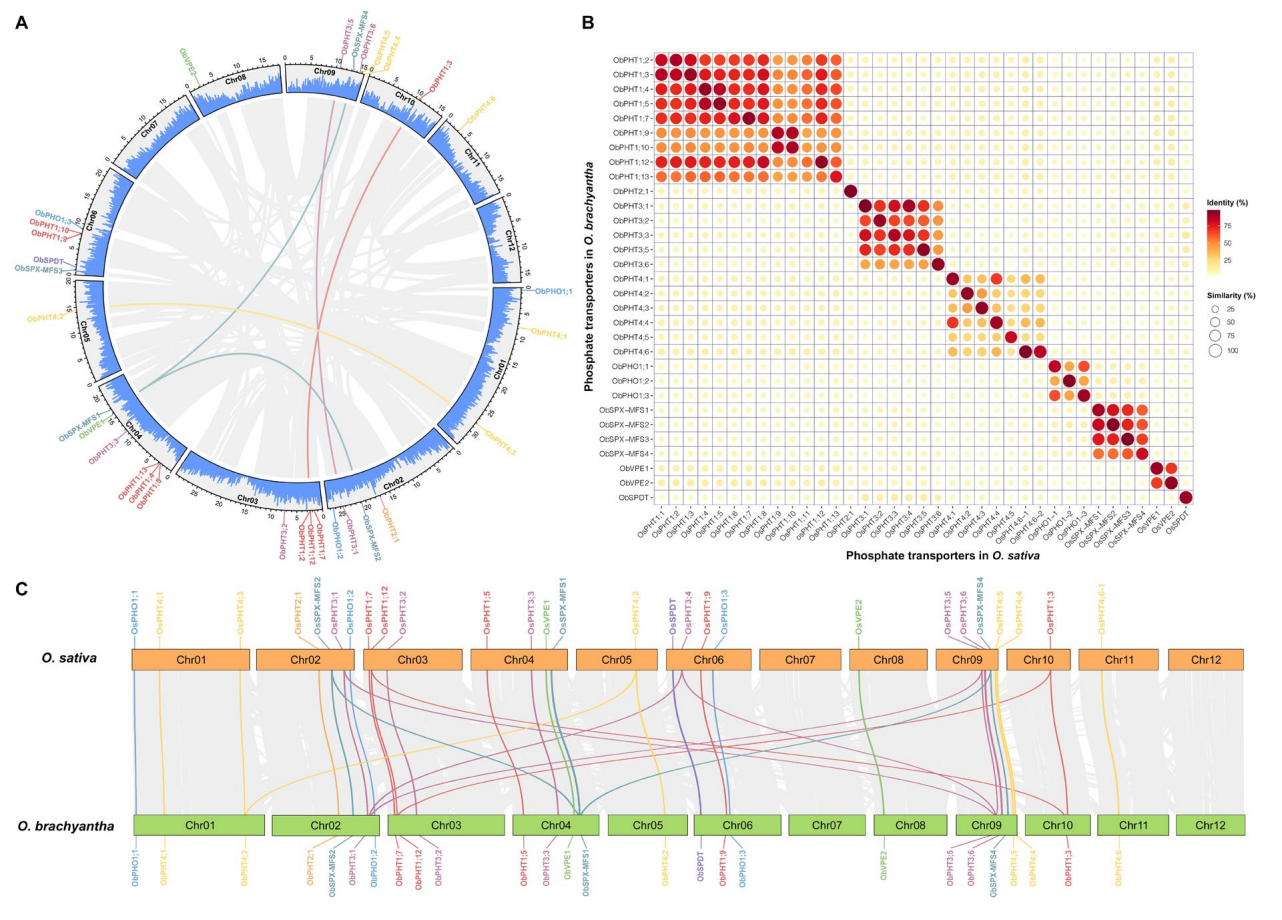
The evolutionary relationships among phosphate transporters (PTs) within the *O. brachyantha* genome and between *O. brachyantha* and *O. sativa* . (A) Chromosomal locations and inter‐chromosomal relationships among *PT* genes within the *O. brachyantha* genome. The gray annulus was drawn in proportion to the lengths of the 12 chromosomes of *O. brachyantha*, and all *ObPT*s were plotted according to their physical distributions in the corresponding chromosomes. Scale bars represent the number of DNA bases in Mb and blue columns on the annulus indicate the gene density of each chromosome in frequency per 0.2 Mb. The chromosome numbers are labeled on the inside of each chromosome. Gray lines crisscrossing between different chromosomes indicate all syntenic blocks in the *O. brachyantha* genome, and the lines connecting syntenic/duplicated *PT* gene pairs are drawn using the same color scheme as in Figures 2 and 3, indicating the *ObPHT1* (red), *ObPHT3* (maroon), *ObPHT4* (yellow) and *ObSPX‐MFS* (dark green) clades, respectively. Other *PT* genes are also labeled using the same color scheme used in Figure 2 to indicate their membership in the eight identified clades. (B) Sequence identity and similarity among all PT proteins between *O. sativa* (Os) and *O. brachyantha* (Ob). The percentage of amino acid sequence identity and similarity are indicated by circle color and size, respectively. (C) Collinearity analyses of *PT* genes between *O. sativa* (Os) and *O. brachyantha* (Ob). The gray lines represent all aligned blocks between the two species, and the syntenic gene pairs within each clade are indicated with the same colors corresponding to the scheme used in Figure 2.

To further characterize the *ObPT* genes, we mapped them to the *O. brachyantha* chromosomes based on their genomic locations using TBtools (Chen et al. 2020). Except for chromosomes 7 and 12, each chromosome contains at least one *ObPT* gene (Figure 4A). There are six *ObPT* genes on chromosome 4, five each on chromosomes 6 and 9, four each on chromosomes 2 and 3, and three on chromosome 1. There are also five duplicated *ObPT* gene pairs, the connecting lines of which are shown in four different colors to indicate their memberships in the *ObPHT1*, *ObPHT3*, *ObPHT4*, and *ObSPX‐MFS* gene groups (Figure 4A). The ratios of the non‐synonymous substitution rate to the synonymous substitution rate (Ka/Ks) range from 0.09 to 0.39, implying that these genes have undergone purifying selections to eliminate deleterious mutations (Table S4). We also compared the protein sequence identity and similarity of the PTs between *O. sativa* and *O. brachyantha* (Figure 4B and Table S5). The dot plot showed that each ObPT most closely resembles its OsPT ortholog in protein sequence, and the highest sequence identity values for each PT are concentrated within the comparison matrix of the same clade, confirming the above phylogenetic results.

Furthermore, we constructed a synteny map of PT families from the *O. brachyantha* and the *O. sativa* genomes to further understand the evolutionary mechanism of the PT families in wild rice (Figure 4C). The FF‐genome type species, *O. brachyantha*, is genetically distant from cultivated rice (which has the AA genome type), and only 70% of the protein‐coding genes of *O. brachyantha* are located in collinear positions to the *O. sativa* genome (Chen et al. 2013). Despite this genetic divergence, 28 out of 31 pairs of homologous *PT* genes between *O. brachyantha* and its cultivated relative were identified as direct syntenic pairs, indicating the remarkable conservation of PTs within the genus *Oryza*, with the conservation level exceeding the average genome‐wide collinear rate.

To explore the selection pressure on *PT* genes after gene duplication, we calculated and analyzed the Ka/Ks ratio of the orthologous *PT* genes between *O. sativa* and *O. brachyantha* (Table S6). Twenty‐eight pairs of orthologous *PT* genes between these two species were obtained by bidirectional Blastp. Their Ka/Ks values ranged from 0.0417 to 0.423, with an average of 0.1785. These values were much lower than 1, suggesting intense purifying selection pressure was experienced during the evolution of *PT* genes within the genus *Oryza*.

### 3.4 Expression Patterns of *ObPT* Genes Under pi Starvation

Given that *O. brachyantha* has a remarkably higher total P content than other *Oryza* species tested in this study, we wanted to investigate in finer detail the responses of the ObPTs to progressive Pi depletion in the root and shoot. Four‐week‐old *O. brachyantha* plants were exposed to Pi starvation for 0, 3, 6, and 12 days, after which the root and leaf tissues were analyzed separately to determine their respective cellular Pi concentrations as well as the temporal and spatial expression patterns of all *ObPT* genes through qRT‐PCR.

**FIGURE 5:**
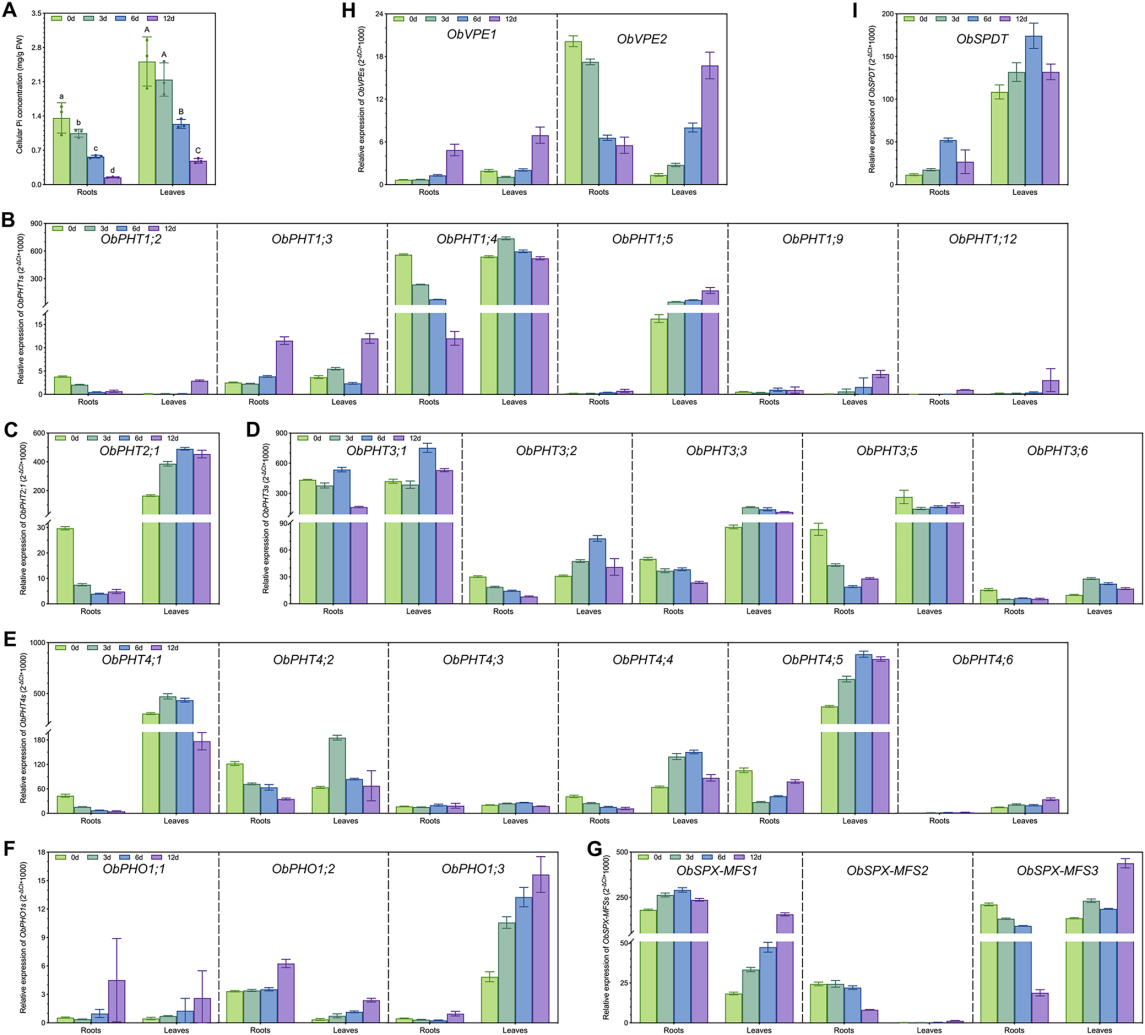
Dynamic changes in the tissue‐specific expression levels of *phosphate transporter* (*PT*) genes in *O. brachyantha* during phosphate deprivation. (A) Concentrations of soluble inorganic phosphate (Pi) in roots and leaves under 0, 3, 6, and 12 d of Pi‐deficient conditions. FW, fresh weight. Different letters within the same tissue indicate significant differences (*p* < 0.05) according to Student's *t*‐test. (B–I) Relative expression analyses of *ObPHT1*s (B), *ObPHT2* (C), *ObPHT3*s (D), *ObPHT4*s (E), *ObPHO1*s (F), *ObSPX‐MFS*s (G), *ObVPE*s (H) and *ObSPDT* (I) after different Pi‐starvation times. All data shown are means ± SD, each with three biological replicates.

Our results showed that Pi concentrations in *O. brachyantha* tissues dropped sharply throughout the 12‐day duration of Pi deficiency treatment (Figure 5A). Cellular Pi values in roots and leaves were reduced by 89.2% and 80.7%, respectively, from the average levels of 1.36 and 2.51 mg g^−1^ FW, respectively, at the beginning of the treatment. Together with the total P results, this shows that *O. brachyantha* may be able to re‐utilize its high P storage to maintain growth for a relatively long period in a Pi‐fluctuating environment.

Our qRT‐PCR results (Figure 5B–I) revealed the diverse expression patterns of 31 *ObPT* genes in response to Pi starvation in different tissues. Among them, four genes (*ObPHT1;7*, *ObPHT1;10*, *ObPHT1;13* and *ObSPX‐MFS4*) were not detected, and three genes (*ObPHT1;9*, *ObPHT1;12* and *ObPHO1;1*) were barely expressed in any tissue, suggesting that they may have limited roles in P homeostasis. On the other hand, we identified four genes that displayed tissue‐specific expression patterns, indicating their potential involvement in Pi acquisition, transport, or storage. For example, *ObPHT1;5*, *ObPHT4;6*, and *ObPHO1;3* were minimally expressed in roots but highly induced in leaves under Pi starvation, while *ObSPX‐MFS2* was barely expressed in leaves and repressed in roots after prolonged Pi starvation.

Moreover, we observed that some genes (*ObPHT1;3*, *ObPHO1;2*, *ObSPX‐MFS1*, *ObVPE1* and *ObSPDT*) were induced by Pi starvation in both types of tissues, suggesting that they may play a universal role in Pi sensing, signaling, or mobilization. Interestingly, most of the remaining *ObPT* genes were downregulated in roots by Pi starvation but upregulated in leaves, suggesting their differential regulations and functions are tissue‐specific. Notably, among these genes, the homologs of *ObSPX‐MFS3* and *ObVPE2* in cultivated rice are responsible for Pi import to and export from the vacuole (Guo et al. 2022; Xu et al. 2019), implying a role in Pi storage and recycling in subcellular compartments. These results provide valuable insights into the distinct roles of different *PT* genes in P homeostasis within different tissues of *O. brachyantha*.

## 4 Discussion

P is an essential macronutrient for plant growth and development, but its availability in the soil is often limited. Fortunately, plants have evolved diverse strategies to cope with P deficiency, such as enhancing P acquisition from the rhizosphere, optimizing P utilization within the plant, and re‐mobilizing P from old to young tissues. Those underlying mechanisms have been extensively investigated in cultivated rice. However, the genetic diversity of domesticated rice is limited compared to its wild relatives in the same genus, and the genetic basis of wild rice adaptations to suboptimal P levels in the soil remains largely unknown. Therefore, we tested a set of wild rice accessions for P absorption efficiency using a hydroponic system. Among the two cultivated subspecies and 12 wild species of rice, *O. brachyantha* exhibited the highest P accumulation under both −P and +P conditions (Figure 1). This trait might confer *O. brachyantha* an adaptive advantage in its natural habitat in the African savanna and suggests a unique efficiency in P use in this wild species with a compact FF‐type genome. As a distant wild relative of cultivated rice in the same genus, *O. brachyantha* represents a valuable resource to explore novel genes for coping with Pi‐limited environments.

Through whole‐genome search using the protein sequences of known *Arabidopsis* and *O. sativa* PTs, we discovered a total of 31 *PT* genes in the *O. brachyantha* genome, including members of the PHT, SPX‐EXS, SPX‐MFS, VPE, and SPDT families. In the phylogenetic tree, all ObPTs were paired with the PTs from cultivated rice, indicating a shared evolutionary history within the genus *Oryza* (Figure 3). Comparisons between the protein sequences of PTs from *O. sativa* and *O. brachyantha* further supported the phylogenetic findings, with each ObPT displaying the highest sequence identity and similarity with its ortholog in domestic rice (Figure 4B). Furthermore, all the Ka/Ks values of homologous PT genes are less than 1, implying that intense purifying selection pressure played a role in maintaining the functionality of these PTs during evolution (Table S6).

However, four OsPHT1s, OsPHT3;4 and OsPHT4;6–2 have no homologs in *O. brachyantha*, suggesting a divergence in the *PT* gene content driven by environmental selection. This indicates that around 19.4% of PTs or 28.6% of PHTs underwent duplication in cultivated rice during artificial selection, which is consistent with the massive amplification of gene families observed in the domesticated rice genome (Gu et al. 2016; Wang et al. 2017, 2020). However, compared to the level of synteny between *O. sativa* and *O. brachyantha* at the genomic level, a higher proportion of *ObPT* genes have synteny relationships with their orthologs in cultivated rice (Figure 4C), suggesting PT proteins are important for survival and therefore are highly conserved during the evolution of the genus *Oryza*.

The expression levels of *ObPHTs* were higher than those of other *ObPT* families (Figure 5), indicating their importance for Pi homeostasis. However, it is intriguing to observe that PHT3s are genetically closer to other PT families than to other PHT subfamilies (Figure 2A and Figure 3). This distinct clustering pattern suggests a potentially close functional relationship between PHT3s and other PT, underscoring the need to examine all PT families together in the comprehensive genome‐wide identification and characterization analysis for other species.

Despite the phylogenetically close relationships between OsPTs and ObPTs, there are notable differences in the dynamics and tissue specificities in their expression patterns in response to Pi fluctuations. In cultivated rice, 10 *PHT1*s have been studied in detail, most of which are involved in Pi uptake and/or translocation. Among the reported *PHT1*s from cultivated rice, *OsPHT1;1*, *OsPHT1;4*, and *OsPHT1;8* are abundantly expressed in different rice tissues and are involved in Pi uptake and allocation independent of Pi supply (Jia et al. 2011; Sun et al. 2012; Zhang et al. 2015). However, out of these four genes, only *OsPHT1;4* has a homolog in *O. brachyantha* (*ObPHT1;4*), which was highly expressed in both roots and shoots, with its root expression decreasing under prolonged Pi‐deficient conditions (Figure 5B). Furthermore, whereas *OsPHT1;2* demonstrates low root expression but high shoot expression (Ai et al. 2009; Liu et al. 2010), *ObPHT1;2* was minimally expressed in both tissues (Figure 5B). Notably, *OsPHT1;3*, *OsPHT1;6*, *OsPHT1;9*, and *OsPHT1;10* maintained low transcript levels in Pi‐sufficient environments but were markedly upregulated in response to Pi deprivation in all root tissues (Ai et al. 2009; Chang et al. 2019; Wang et al. 2014). However, in *O. brachyantha*, this expression pattern was predominantly mirrored by *ObPHT1;3* only, with *ObPHT1;9* displaying subdued expressions and *ObPHT1;10* remaining undetectable in both tissues (Figure 5B). Additionally, *OsPHT1;11/13* represent two symbiosis‐specific members essential for the establishment of symbiotic interactions between arbuscular mycorrhizal fungi and plant roots (Paszkowski et al. 2002; Yang et al. 2012), whereas only a single symbiosis‐specific homolog, *ObPHT1;13*, is present in *O. brachyantha* but remains unexpressed in roots. These findings imply a possible divergence in the functions of the orthologous *PT* pairs between *O. sativa* and *O. brachyantha*, despite their close genetic distances.

The expression dynamics of other *ObPT* genes under Pi starvation also revealed tissue‐specific and context‐dependent regulations. For example, *ObPHO1;2* (Figure 5F), *ObSPX‐MFS1* (Figure 5G), *ObVPE1* (Figure 5H), and *ObSPDT* (Figure 5I) showed induction across both tissue types in response to Pi scarcity, implying their putative roles in Pi sensing, signaling, or mobilization. Interestingly, a majority of the remaining *ObPTs* were downregulated in roots but upregulated in leaves under Pi starvation, indicating their diverse functions in Pi homeostasis within different tissues. Taken together, these dynamic expression patterns support the scenario of the efficient re‐utilization of high P storage in roots and leaves for an extended period, potentially explaining its superior Pi utilization strategy. Further investigation of the functional roles of specific ObPTs and their potential applications in crop improvement will contribute to the development of more efficient and sustainable agricultural practices.

## 5 Conclusion

The distinct Pi uptake efficiency observed in *O. brachyantha* coupled with the conservation and diversification of *PT* genes highlights the intrinsic potential of this wild rice species as a valuable genetic resource for improving P utilization in crops. Moreover, expression profile analyses showed that different *ObPT*s displayed differential expression patterns under P scarcity, suggesting that they may have different roles or regulatory mechanisms in Pi homeostasis. This study provides a foundation for future breeding and engineering strategies to enhance Pi acquisition and P use efficiency in cultivated rice and other crops.

## Author Contributions

**Hon‐Ming Lam:** conceptualization (lead), funding acquisition (lead), project administration (lead), supervision (lead), writing – review and editing (lead). **Xusheng Zhao:** data curation (lead), investigation (lead), methodology (lead), software (lead), visualization (lead), writing – original draft (lead). **Wai‐Shing Yung:** funding acquisition (supporting), project administration (supporting), supervision (supporting), writing – review and editing (supporting). **Kejing Fan:** conceptualization (supporting), supervision (supporting), writing – review and editing (supporting). **Shengjie Chen:** data curation (supporting), investigation (supporting), writing – original draft (supporting).

## Conflicts of Interest

The authors declare no conflicts of interest.

## Supporting information


**Table S1.** Information on the *ObPT* primers used in qRT‐PCR assays.
**Table S2.** Gene names and locus IDs of all phosphate transporter genes used in phylogenetic analysis.
**Table S3.** Detailed properties of phosphate transporters identified from the *Oryza brachyantha* genome.
**Table S4.** Comparisons of the substitution rates among homologous phosphate transporter (PT) genes within the *O. brachyantha* genome.
**Table S5.** Amino acid sequence identity and similarity between all the phosphate transporter (PT) proteins of *O. sativa* and those of *O. brachyantha*.
**Table S6.** Comparisons of the substitution rates of homologous phosphate transporter (PT) genes between the *O. brachyantha* and *O. sativa* genomes.

## Data Availability

The data that supports the findings of this study are available in the Supporting Information of this article.
